# Design and Implementation of Survey Quality Control System for Qatar’s First National Mental Health Survey: Case Study

**DOI:** 10.2196/37653

**Published:** 2023-11-27

**Authors:** Catalina Petcu, Ikram Boukhelif, Veena Davis, Hamda Shamsi, Marwa Al-Assi, Anis Miladi, Salma M Khaled

**Affiliations:** 1 Social and Economic Survey Research Institute Qatar University Doha Qatar

**Keywords:** World Mental Health Survey, quality control indicators, Middle East, phone interview, case study, COVID-19

## Abstract

**Background:**

All World Mental Health (WMH) Surveys apply high standards of data quality. To date, most of the published quality control (QC) procedures for these surveys were in relation to face-to-face interviews. However, owing to the social restrictions that emerged from the COVID-19 pandemic, telephone interviews are the most effective alternative for conducting complex probability-based large-scale surveys.

**Objective:**

In this paper, we present the QC system implemented in the WMH Qatar Survey, the first WMH Survey conducted during the COVID-19 pandemic in the Middle East. The objective of the QC process was to acquire high data quality through the reduction of random errors and bias in data collection.

**Methods:**

The QC design and procedures in this study were adapted to the telephone survey mode in response to the COVID-19 pandemic. We focus on the design of the QC indicator system and its implementation, including the investigation process, monitoring interviewers’ performance during survey fielding and applying quality-informed interventions.

**Results:**

The study team investigated 11,035 flags triggered during the 2 waves of the survey data collection. The most triggered flags were related to short question administration duration and multiple visits to the same survey questions or responses. Live monitoring of the interviews helped in understanding why certain duration-related flags were triggered and the interviewing patterns of the interviewers. Corrective and preventive actions were taken against interviewers’ behaviors based on the investigation of triggered flags per interviewer and live call monitoring of interviews. Although, in most cases, the interviewers required refresher training sessions and feedback to improve their performance, several interviewers discontinued work because of low productivity and a high number of triggered flags.

**Conclusions:**

The specific QC procedures implemented in the course of the WMH Qatar Survey were essential for successfully meeting the target number of interviews (N=5000). The QC strategies and the new indicators customized for telephone interviews contributed to the flag investigation and verification process. The QC data presented in this study shed light on the rigorous methods and quality monitoring processes in the course of conducting a large-scale national survey on sensitive topics during the COVID-19 pandemic.

## Introduction

### Background

Survey quality is determined by the accuracy, reliability, and validity of data [1]. Measures implemented to acquire high data quality define the quality control (QC) process, which aims at the detection and reduction of errors in data collection as well as the prevention of practices that do not follow the study protocol. QC is defined as planned efforts to monitor, verify, and analyze the quality of data as it is being collected, thus enabling continuous quality improvement during data collection [2]. The World Mental Health (WMH) Surveys implement a high standard of QC [1,3-6] to reduce errors and unacceptable practices, including falsification in the data collection. In several countries where the WMH Survey was conducted, such as the United States, China, Germany, Lebanon, New Zealand, and Spain, data falsifications have been reported [1,4]. Falsifications included making up all or part of an interview, miscoding the answer, reporting the wrong case disposition, and interviewing a nonsampled individual [7]. Therefore, the implementation of QC procedures plays an important role in conducting complex community-based surveys.

To date, WMH Surveys have been conducted in >30 nations, including Arab countries such as Lebanon, Iraq, and Saudi Arabia [8-14]. Scholars have presented global perspectives on the epidemiology of mental disorders and provided detailed information about QC in some of the WMH studies [15]. However, the QC procedures for most of these surveys were in relation to in-person interviews or face-to-face survey mode. Moreover, most of these studies were based on the paper-and-pencil version of the Composite International Diagnostic Interview (CIDI) and not on the computer-assisted personal interviewing (CAPI) version of the CIDI. CIDI was designed by the World Health Organization to assess for psychiatric disorders and associated risk factors, and treatment interventions. The latter version of the CIDI was a greater improvement than the original paper-and-pencil version, which was much longer to administer and was more prone to skip logic and data entry errors. Furthermore, administration using computer technology necessitated a different form of QC procedures. Nevertheless, several published papers from the WMH countries that used CAPI technology focused mainly on survey methodology and implementation, whereas QC processes or procedures were rarely mentioned [6,9,16,17]. QC of computer-assisted telephone interviewing (CATI) operations in Canada [18] tracked and evaluated the interviewer’s performance using quality indicators. In 1996, Mudryk et al [18] identified the interviewer’s problems and explained the quality procedure for CATI implementation and methodology. The Singapore Mental Health Study [19] demonstrated the design and methodology of the research, including quality of survey administration, field staff selection, productivity, and data analysis. There is a short paragraph in the Iranian Mental Health Survey [17] regarding QC of the fieldwork.

In the Middle East, large-scale mental health surveys rarely report the implementation of QC measures or procedures. However, scholars recommend the adoption of efficient QC tools and the development of strong teams to manage QC procedures as a priority [2,20]. To date, the most recent and comprehensive information on the development and implementation of the QC system was provided for the WMH Survey conducted in Saudi Arabia using CAPI mode [21]. In this study, we focused on the different phases of the QC cycle from the quality management perspective of the survey process. A study on the challenges and lessons from piloting the WMH Survey in Qatar described the development and implementation of a QC system to monitor interviewing activities in the field during the face-to-face survey pilot of the WMH Qatar study [22].

In this paper, we describe how we created and implemented a QC system for the first national mental health survey in Qatar, the WMH Qatar Survey, which is also one of the first studies in the WMH Survey consortium conducted using CATI technology during the COVID-19 pandemic. The QC design and procedures in our study were also adapted to the CATI mode of data collection. In other parts of the world, a study investigating the prevalence of suicidal thoughts and behaviors in the Spanish adult general population during the first wave of COVID-19 also used the CATI survey mode [23], whereas other studies collected data using web surveys [24-28]. However, none of these recent studies have focused on QC procedures.

This study provides an overall framework for implementing similar QC procedures in future large-scale population surveys, including the WMH Surveys using the CATI methodology. First, we briefly present information on the sampling design and fieldwork operations and procedures. Then, we discuss in detail the design of the QC system, implementation of quality indicators, flags investigation process, interviewers’ performance monitoring, and QC-informed interventions implemented during the fielding of the survey. At the time of writing this paper (ie, January 2022), the production and the QC process were in the final phase.

### Objectives

This paper aims to (1) describe the design of the QC system adapted to the CATI mode of data collection, (2) discuss the QC measures that have been implemented to detect errors in data collection and prevent practices that do not follow the study protocol, and (3) provide a framework for developing QC systems and procedures in future large-scale population surveys.

## Methods

### Sampling Design

The target population of this study included Arab adults aged ≥18 years living in Qatar during the survey reference period (ie, from January 2020 to January 2021). To reach this population, the Social and Economic Survey Research Institute (SESRI) worked with local cellular phone providers to develop a cellular phone frame. A probability sample was drawn from this frame by using the listed dialing technique. The proportion of adults with a cellular phone in Qatar is approximately 98% and it was determined by a SESRI statistician for the National Omnibus Survey conducted in 2018. A sample drawn from this type of frame was expected to have excellent coverage and representation of the target population. Because the target population could not be completely identified in the frame, we had to oversample certain groups of phone numbers that were likely to belong to the target population. This oversampling was also important to avoid sampling nonworking phone numbers. The sampled phone numbers were released for interviewing in batches to ensure that the complete call procedures were followed for all phone numbers. The phone calls were made at different times during the day and on different days of the week to maximize the chances of making contact with potential respondents.

### Fieldwork Operational Team and Procedures

#### Overview

The fieldwork team and data collection played an essential role in the QC process. The interviewers recruited for this study were trained on the survey administration protocol and data capture software. In the following sections, we present details of the training and data collection procedures, including the software used and the changes in the phone interviewing approach necessitated by the COVID-19 pandemic.

#### Interviewers Training

In interviewer-administered surveys, the training is considered an important part of the QC [29]. Conducting this large-scale survey by phone required a rigorous training program for the survey interviewers. To improve the performance of existing and newly recruited interviewers, training sessions were organized with the aim of enhancing the interviewers’ reading, probing, persuading, and IT skills. The training was conducted for >5 days for 4 to 5 hours each day.

A total of 3 teams were involved in the training process: the research team, the CATI laboratory team, and the IT team at SESRI, Qatar University. The training materials included frequently asked questions sheet to support answering the respondents’ questions and concerns regarding the study. The respondent booklet contained survey response options, whereas the interviewer booklet listed scenarios and instructions for the interviewers’ reviews and references, as well as scripts that were used during the practical part of the interviewers’ training. During the training sessions, the interviewers were familiarized with the questionnaire, including diagnostic and nondiagnostic modules of the CIDI. The importance and aims of the study were highlighted, as well as media coverage of the study. General interviewing techniques (eg, reading questions, probing, accurately entering respondent’s responses and comments, and addressing soft and hard refusals) were also covered by the CATI laboratory team during the training, in addition to the study’s interviewing protocol and guidelines. The training also covered information about the importance of adherence to the ethical standards of scientific research, including informed consent and confidentiality. The IT team demonstrated the use of Blaise software (CBS Statistics) for entering data and trained the interviewers on IT-related procedures. Participants in the training program who had the best performance on the scored tasks during the training were selected to become interviewers in the WMH Qatar study.

#### CATI Laboratory Organizational Structure

The CATI team comprised a manager, an assistant data collection specialist, 6 supervisors, and 42 interviewers. The interviewers worked 1 of 2 shifts (ie, 9 AM to 3 PM and 3 PM to 9 PM) every day of the week from Sunday to Saturday.

#### Data Collection

CATI is a telephone interviewing method in which interviewers use an electronic device, specifically a tablet, with Blaise data entry client software to read the survey script and enter the information collected and with Cisco Communicator software (Cisco Systems) to make the calls [30]. The survey used CATI to administer the CIDI 5.0 instrument during the 2 waves of production. The first wave commenced on January 20, 2021, until April 12, 2021, whereas the second wave was conducted from May 25, 2021, to July 15, 2021, and continued after the summer break from September 19, 2021, to January 2022.

The data collected were automatically sent in real time to the SESRI central server using the Case Management System (Cisco Systems). Because of Coronavirus restrictions, interviewers conducted their activities from home. Remote work required each interviewer to connect to the Global Protect Virtual Private Network (Palo Alto Networks) [31] to ensure a secure connection and to protect data transfer to Qatar University’s servers against mobile threats. Using such computerized data collection methods ensures the following: reducing item-level missing data, obtaining timely data, and collecting process data or paradata. Paradata included process data, such as call records, interviewer observations, time stamps, and call dispositions.

Because of the restrictions imposed on face-to-face interviews during the COVID-19 pandemic, it was decided to move CATI survey operations to a distributed CATI mode, as opposed to a centralized call center. In this data collection mode, because interviewers worked from home, they tended not to adhere to their working hours. Furthermore, supervisors were not able to directly monitor interviewers as they worked. To fill this gap, the SESRI IT team developed an app called CATI Time Tracker that allowed interviewers to log their work activities and breaks throughout their working shifts, which were accessible to CATI managers and supervisors. The CATI Time Tracker app also facilitated shift management and interviewer evaluations for supervisors, as well as provided technical support. The data generated through the app were fed into the QC database, which were used to monitor predefined QC indicators during the data collection phase of the study.

### Ethical Considerations

This study was approved by Qatar University Institutional Review Board. The Research Ethics Approval Number is QU-IRB 1219 EA/20. As the survey was conducted using the telephone mode of interviewing, verbal informed consent to participate was obtained from all study participants. To safeguard against potential risks of participation, standard research protocols and the Health Insurance Portability and Accountability Act were followed. All respondents were assigned anonymous study codes for identification, and only aggregate data were used.

### QC Indicator System

#### Overview

The QC database mentioned above was a key component of the QC indicator system (QCIS), which transformed, integrated, and aggregated data into tables that store information on various indicators, allowing updates on the progress of data collection through visualizations used by the QC team to investigate the performance of the interviewers. To holistically comprehend the QC system, we present and discuss in this section the components of QCIS, modifications implemented to adapt the QC process to the phone interviewing mode, and the metrics and indicators, also called flags, that the QC team refers to when investigating, monitoring, and correcting interviewers’ performance and behavior.

#### Server Infrastructure and Data Components

The data collected by the interviewers and paradata were categorized into different sources that were fed into the QC system. Through different schedules of frequency processing, quality indicators objectively measured the efficiency of the essential segments of data collection. Quality indicators were vital in the QC process because they offered the possibility of rapid insight into the quality of the collected data, the performance of the interviewers, and their patterns related to their performance with time [32].

QCIS was developed by the SESRI IT team for the WMH Qatar study. The foundation of the QC tool was initially established for the CAPI survey mode in collaboration with the University of Michigan team. Switching to CATI mode involved major changes to the QC tool implemented by the SESRI IT team; these changes are presented below. In developing the QC tool, the SESRI IT team identified variables for QC indicator flags, created scripts to generate the indicator flag, designed a schema to store flag information and desired aggregated data, designed a periodic QC indicator processor to update the flag data in the Master QC, and developed sample charts and tables to use as visualizations of the QC indicators.

**Figure 1:**
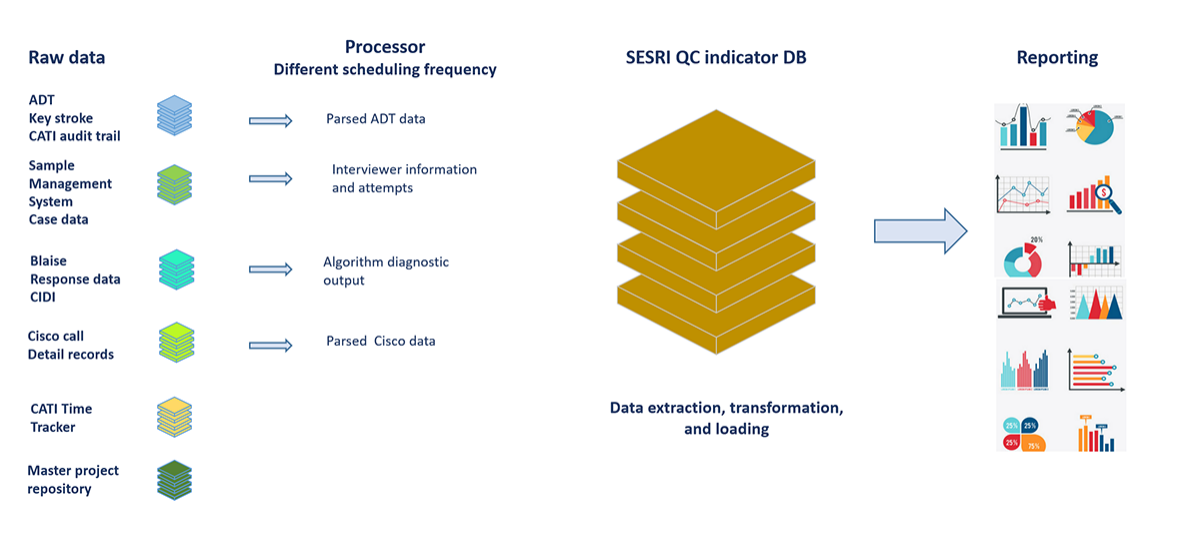
Quality control indicator system designed for World Mental Health Qatar Study by Social and Economic Survey Research Institute (SESRI) IT team. ADT: audit trails; CATI: computer-assisted telephone interviewing; CIDI: Composite International Diagnostic Interview; DB: database; QC: quality control.

Data coming from different sources were loaded into the QC database. Before the pandemic, when the interviewing mode was face-to-face, the QC infrastructure had the following sources: CATI audit trail (ADT), Sample Management System, and Blaise Response Data CIDI. These sources were processed into the QC database, and using the SQL analysis service to transform and the server analysis services tabular model to aggregate, the indicators were reported through charts, tables, and graphs on the Power Business Intelligence (Power BI; Microsoft Corp) dashboard. The structural changes implemented by the SESRI IT team because of the new mode of interviewing involved the addition of 3 new sources: Cisco call detail records (CDR), CATI Time Tracker, and Master Project Repository. Moreover, the server analysis services model was removed, and the data extraction transformation and loading process was integrated within the QC database (Figure 1).

For a better understanding of the raw data fed into the QC database, a brief description of the data sources will provide insights into the information used for developing indicators. The 6 data sources are explained as follows:

1. CATI ADT was generated by the Blaise software as interviewers went through the interview. ADT data were parsed daily and included information on Blaise sessions, all activities in survey fields, including start and end timestamps, and keystrokes. This information was used when flagging for survey time–related QC indicators.
2. The Sample Management System used a call scheduler, which was responsible for scheduling telephone numbers in the day batch, making the cases available to the interviewers at the scheduled time.
3. Blaise response data were also consumed by the QC system, including the start and end timestamps of the call, call outcome, appointment details, case status, respondent type, and gender. The Blaise data were collected in near real time (delays of a few seconds or minutes) and were vital for performing QC and for monitoring the progress of the study.
4. CDR data provided a record of all calls that have been made or received by users of the Cisco Call Manager system and were useful for tracking call activity and monitoring sessions. CDR data were collected by SESRI, filtered to keep only outgoing calls from SESRI phone numbers, and transformed to extract details such as call start and end timestamps, duration of the call, correct phone number, and monitoring sessions that occurred during the call, if any.
5. The CATI Time Tracker was also an important source that provided details about interviewers’ activities. The Time Tracker helped the data collection agents keep track of how each interviewer’s workday was distributed on different predefined activities such as calling, shift preparation, and technical support. These data were compared with the agent’s Blaise activity to check for underreporting or overreporting of work hours.
6. The Master Project Repository was a database that served as the basis for the payroll system. It included data such as phases of studies conducted by SESRI, employee details, agents participating in each study and their roles, past payment details, and history of agent employment.

These data sources underwent preprocessing, including filtering, parsing, and reshaping. This was done daily through special scripts and run using TeamCity (JetBrains) software. After preprocessing, the data were loaded into the QC database, which further transformed, integrated, and aggregated data into tables that stored information on dials, interviewer activities, values for different metrics and indicators, and the status of cases. This process was done using a series of specialized procedures that ran consecutively on SQL Server Management Studio [33] and initiated by TeamCity every 15 minutes throughout the day. This process allowed for the updated progress of data collection to be seen as visualizations on the Power BI dashboard.

#### Indicators or Flags in Power BI

The visualizations on the Power BI dashboard were based on indicators that have been attentively selected and implemented to reflect the requirements of the phone interviewing mode. The SESRI IT team and the research team met regularly to discuss the indicators required for the new mode of interviewing.

The Michigan team assisted in the implementation of these modified flags. After reassessing the old indicators for the CAPI mode, a few of the face-to-face mode indicators were kept with new cutoffs, and new indicators were developed by the SESRI IT team and displayed on the Power BI dashboard (Textbox 1). The initial cutoffs were determined by the team at Michigan University and were used in previous WMH face-to-face surveys. Nevertheless, when the interviewing mode of the WMH Survey in Qatar changed to telephone mode owing to COVID-19 social restrictions, the cutoff values of the CAPI mode indicators were no longer applicable. The IT team at SESRI implemented new cutoffs based on the features of telephone interviewing. For example, the shorter length of phone interviews compared with face-to-face interviews lead to different cutoff value for the *short average interview length* flag. The country’s cultural setting was also considered when determining the cutoff values for some of the flags. For example, prayer time affected the cutoff values used for the *long question field time* and *long interview length* flags. The pilot phase of the study provided an important opportunity to further test and adjust these values before the survey production phase.

To facilitate flag identification by each team, the indicators were categorized into CIDI-related indicators, duration-related indicators, IT indicators, and operational indicators. This categorization allowed each team to focus on the flags assigned for review and verification. In addition, the IT team developed new metrics to verify whether the interviewers were adhering to their working hours and to collect monitoring details (Textbox 2). This was of great importance as the interviewers were working from home, and the supervisors were not able to directly monitor the interviewers as they did in the centralized CATI system setup. Quality indicators were one of the tools used to monitor and control process functioning, whereby the data collected provided a basis for the implementation of corrective measures and continuous quality improvement activities.

**Figure 2:**
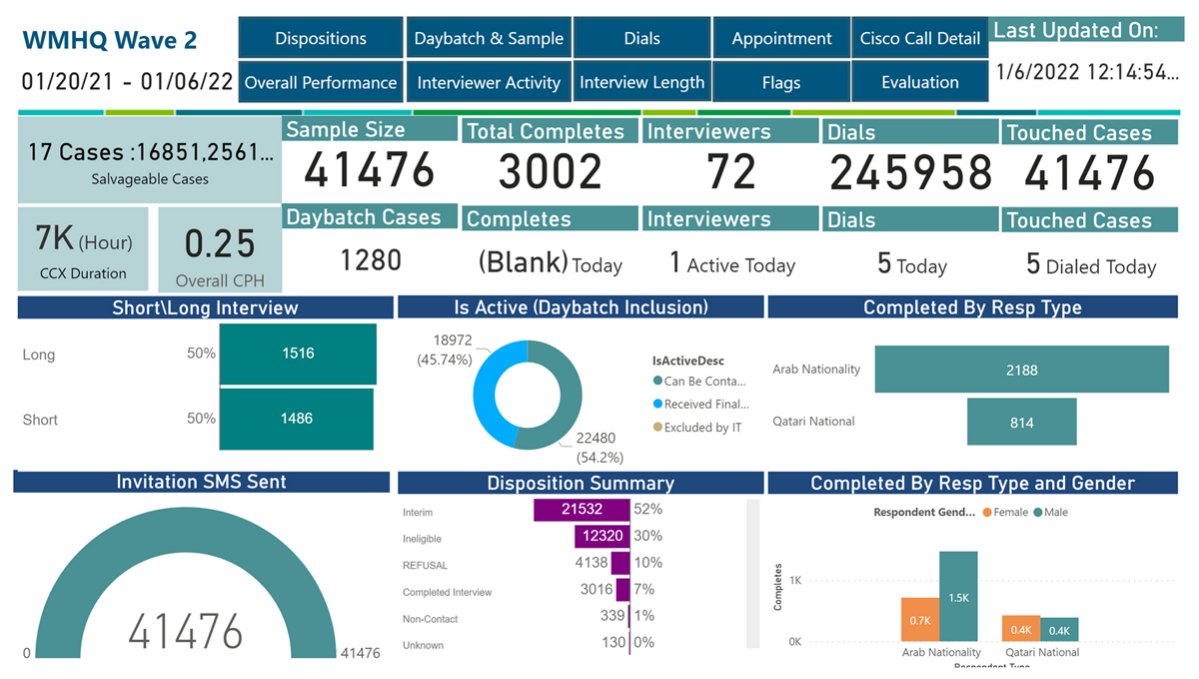
Power Business Intelligence dashboard—main page displaying metrics of the World Mental Health Qatar Survey.

After discussing with the research team, the IT team, prioritized the information to be displayed on the first page of the Power BI dashboard. This provided a summary of the progress of interviewers that the field operations and the research teams were able to access in a fast way. The overall number of completed interviews per hour and the total duration of all Cisco calls were implemented on the dashboard, as well as a chart of the percentages of long and short completed interviews. Other indicators displayed on the main page were total sample size, sampled cases, total completed interviews, completed interviews per day, total number of interviewers, number of active interviewers per day, total number of dials, number of dials per day, disposition summary, and respondent type and gender (Figure 2).

Other tabs in the Power BI dashboard include visualizations of dispositions, overall performance, Cisco call details, interviewer activity, evaluation, and appointments. Another page containing all the flags by the interviewer provided a holistic perspective on the overall performance of the interviewers and enabled the users to filter the information by date, interviewer, category, and type of flags, including the flags calculated for a period of 2 weeks, called *z* score flags.

All the mentioned flags and metrics were displayed on the Power BI dashboard via graphs, charts, tables, and other infographics. The Power BI dashboard was customized for the WMH Qatar study, which helped the research team maintain oversight of survey progress and adherence to the protocol. The indicators were automated and updated daily, enabling live updates on productivity and interviewers’ performance in the field.

**List of quality indicators or flags developed for World Mental Health Qatar phone survey.**

**List of flags**

- High number of completes
- Short question field time
- Short stem question field time
- High percentage of short field time
- Short interview length
- Long interview length
- Short average interview length
- High number of negative stems
- Multiple field visits
- Multiple stem-field visits
- Low prevalence rate
- Long question field time
- Long treatment length

**Verification metrics developed by the Social and Economic Survey Research Institute IT team for the World Mental Health Qatar phone survey.**

**Metrics developed by the Social and Economic Survey Research Institute IT team**

- Monitored
- Monitoring time per case
- Monitoring time per interviewer
- Blaise survey time
- Blaise adjusted total time
- Blaise treatment time
- Cisco calling time
- Average after call work time
- Dials per hour
- Number of completions per hour
- Adjusted number of completions per hour
- Discrepancy in reported working time
- Audit trail section length
- Blaise timeout bug flag

### QC Operational Team and Procedures

#### Overview

For the WMH Qatar Survey, QC procedures included automated procedures applied to the collected data through the QC tool, manual verification and investigation of the indicators, prioritizing and filtering the most concerning flags, monitoring the performance of the interviewers, evaluating performance and progress, and applying corrective and preventive measures to improve interviewers’ performance. The composition of the QC team and the details of each QC procedure are presented as follows.

#### Organization of QC Team

The QC research team was formed of the QC supervisor who oversaw the QC process from the research side and investigated the flags, another flag investigator from the IT team, and 4 phone monitors in charge of evaluating the interviewers during the interviews. Moreover, 5 supervisors from the CATI laboratory monitored and evaluated the interviewers.

#### Verification and Flags Investigation Process

The process of reviewing the flags served 4 main objectives: (1) obtaining information about the interview (eg, length, attempts, revisiting questions, and duration of the questions), (2) providing information about the overall data quality of the survey (eg, low prevalence and a high number of negative stem questions), (3) detecting and preventing data falsification, and (4) improving interviewer performance by reinforcing the requirements to fall into specific parameters.

**Figure 3:**
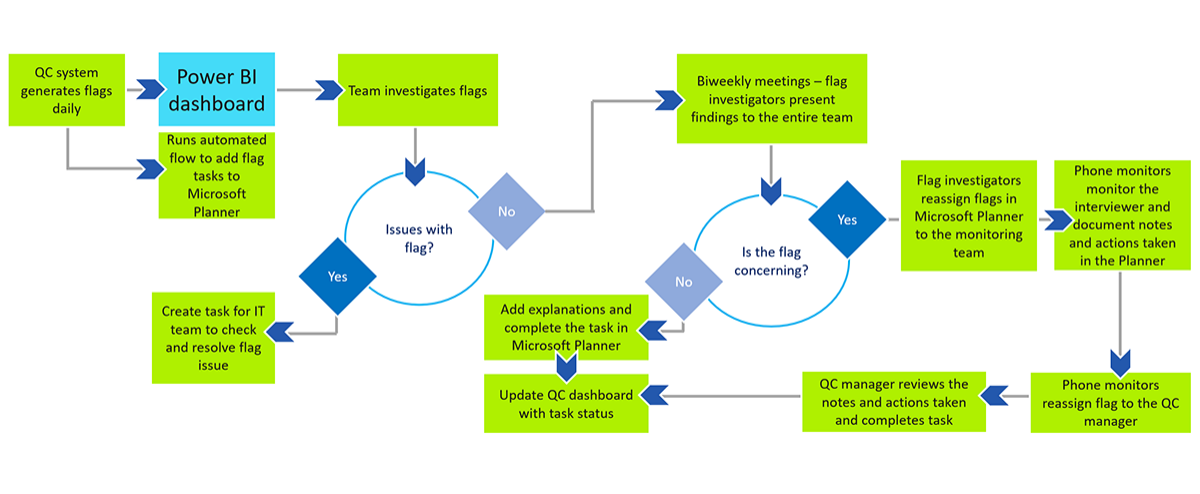
Quality indicators verification and investigation process. Power BI: Power business intelligence; QC: quality control.

Several steps pertained to the verification and investigation processes (Figure 3). The QC system generated flags daily; these flags were loaded on the Power BI dashboard and as tasks on the Microsoft Planner (a program that is used to document the notes and actions taken for each flag). The team started investigating the flags by exporting from the dashboard the table of flags triggered within a specific interval (usually every 3 days) by each interviewer. The initial step was to determine whether the flag was valid or not and if there were technical issues. In the presence of any technical issue (eg, discrepancies, erroneous values—999, and duplicated *z* scores), a task was created for the IT team to check and resolve the flag issue. If there were no technical issues identified, the flags were compiled in an Excel (Microsoft Corporation) sheet with details, notes, and comments and presented to the entire QC research team during biweekly meetings. It was crucial to prioritize and filter the flags according to their level of gravity. The flag investigator determined, based on the findings of the examination, whether a flag was concerning or not. If a flag was concerning, it was reassigned to the monitoring team in the Planner for further investigation. In addition to assigning flags to the Planner, the QC supervisor sent an email at the end of each biweekly meeting with a list of interviewers who required further monitoring and evaluation of their skills. Then, the monitoring team observed the interviewers, added notes and actions in the Planner, and reassigned the task to the QC supervisor to review the added notes. If the explanations and actions were satisfactory, the task was recorded as completed, and the dashboard was updated with the task status.

#### Monitoring the Interviewers

Monitoring allowed us to investigate further suspicious patterns of interviewers’ behaviors and aided us in understanding why certain flags were triggered. The QC staff monitored the progress of the survey activities. Most WMH Surveys recommended 1 monitor for every 8 to 10 experienced interviewers [34]. The monitoring team identified where mistakes occurred during the interview and under what circumstances these mistakes took place and provided insight into respondents’ behaviors as well [35].

The interviews were monitored live (not recorded) by members of the research team and CATI laboratory supervisors using the Cisco Finesse software (Cisco Systems). The monitors looked for specific anomalies according to the concerning flags assigned by the QC supervisor in the Planner. For instance, if an interviewer was flagged for having a short question field time, the phone monitor observed the reading speed and verified whether this particular interviewer was reading the questions verbatim as per the protocol or not.

#### Monitoring Load

The entire duration of all dials made by the interviewers was 9767 hours. We defined dial duration as a telephone connection that starts when the respondent answers the incoming phone call. The monitoring duration accounted for 6.01% (586/9767) of the total duration of dial time. Looking at the completed cases only, 12.08% (543/4496) of them were monitored for at least 15 minutes. Approximately 21% (944/4496) of the completed cases were monitored for at least several minutes (including the cases monitored for <15 min and >15 min).

#### Evaluation

The monitoring team evaluated the interviewers by scoring their skills on the Power BI dashboard and CATI Time Tracker. The following skills were evaluated: probing techniques, dealing with objections and refusals, ability to persuade participants to participate in the survey, typing speed, project knowledge and the ability to answer questions and concerns, and reading the question verbatim without extra explanation or personal opinions. The interviewers’ working hours and the number of completed interviews were also verified. Each skill was scored on a scale from 0 (lowest) to 5 (highest). Monthly and biweekly vouchers were offered to interviewers who adhered to the study protocols, triggered fewer flags, and showed progress after receiving feedback.

#### Interventions

Monitoring notes revealed that most of the flagged interviewers were reading the questionnaire in a rapid manner, skipping words, pronouncing words unclearly, and providing interpretations. Such behaviors required both preventive and corrective measures to reinforce the importance of interviewing protocols and guidelines. The preventive actions included refresher sessions, observing the interviewer’s performance for anomalies, reminding the interviewers to follow fieldwork protocols, and using the interviewer booklet for clarifications. Corrections were made by supervisors and phone monitors by contacting the interviewers to provide feedback and instructions. In case of lack of improvement, further action was taken by the CATI laboratory manager by sending a final warning to the interviewers who failed to show any progress.

## Results

From the beginning of fielding until the moment of writing this paper, the QC team investigated the flags, focusing on the low prevalence rate, negative stems, number of completed interviews per day, and duration of the interview and questions. The team investigated 11,035 flags triggered during the 2 waves of survey production.

Table 1 lists the number of flags triggered by category. The highest number of flags triggered among all the indicator categories is for the *multiple stem-field visits* flag (4584/11035, 41.54%). A high percentage of multiple visits by interviewers was observed for the stem questions in the stressful experiences module (2751/4584, 60.03%), depression module (1243/4584, 27.12%), and worry and anxiety module (277/4584, 6.05%). With a cutoff of 1 visit per stem question, most stem questions were visited 2 times (3300/4584, 72%) and 3 times (1019/4584, 22.24%). The highest number of stem-field visits was 13 for a question in the stressful experiences module.

**Table 1:** Number of quality flags triggered during the World Mental Health Qatar Survey fielding (total number of flags=11,035).

Flags	Flags, n (%)	Cases flagged, n (%)	Total cases, n
High number of completes	50 (0.45)	226 (5.02)	Completed cases: 4496
High number of negative stems	20 (0.18)	20 (0.44)	Completed cases: 4496
High percentage of short field time	181 (1.64)	181 (4.02)	Completed cases: 4496
Long interview length	95 (0.86)	95 (2.11)	Completed cases: 4496
Long treatment length	12 (0.1)	12 (0.02)	All touched cases: 54,850
Multiple field visits	1926 (17.45)	770 (17.12)	Completed cases: 4496
Prevalence rate	46 (0.41)	345 (7.67)	Completed cases: 4496
Short interview length	268 (2.42)	268 (5.96)	Completed cases: 4496
Short question field time	3224 (29.21)	1400 (2.55)	All touched cases: 54,850
Short stem question field time	422 (3.82)	272 (0.49)	All touched cases: 54,850
Multiple stem-field visits	4584 (41.54)	1902 (42.3)	Completed cases: 4496
Long question field time	193 (1.74)	180 (0.32)	All touched cases: 54,850

Out of the total number of 1926 flags of multiple field visits for nonstem questions (Table 1), the highest percentages per module were 20.06% (386/1926) in the stressful experiences module, 20.32% (391/1926) in the health module, and 10.4% (200/1926) in the Covid-19 section. With a cutoff of 3 visits per nonstem question, most of the nonstem questions were visited 4 times (1213/1926, 63.02%) and 5 times (484/1926, 25.13%). The highest number of visits were 24 and 34 for the same question about confirming age and residency in the phone introduction section.

The QC team investigated multiple field visits for stem and nonstem questions, focusing on whether the interviewers were changing to negative answers to minimize the duration of the interview. In most cases, the answers were not modified, and when interviewers were monitored or contacted to provide explanations, it was found that respondents wanted to revisit the questions because they did not understand them. Other motives, such as call interruptions, rapid entry of the option before respondents finalized answering, and respondent’s request to go back to a module at the beginning of the survey, explained revisiting questions. Another motive was the sensitive nature of some questions in these modules, which may have prompted the readdressing of questions. This finding also explains why the module with the most multiple field visits (stem and nonstem) is the stressful experiences module, which contained questions about traumatic situations, including sexual harassment and rape.

The short question field time was the second most triggered flag (3224/11035, 29.21%). The research team tested the questionnaire and verified which questions were <3 seconds by default and eliminated those from the questions to be flagged. Upon further monitoring, the interviewers who read the questions in <3 seconds and were flagged, we found that they were reading the questions in a rapid manner, skipping words, selecting the option before the respondent gave a final answer, and even not reading verbatim but using their own words. Corrective actions for such behavior included reinforcing instructions through SMS text messages or phone calls by a member of the monitoring team. An example of a corrective message to the interviewer was as follows: read the questions clearly at a medium speed (especially the stem questions), read the full answer options if applicable, and record the answer after the respondent’s response, not while still talking. Some of the preventive actions included the supervisor and the monitoring team keeping an eye on a particular interviewer’s performance for anomalies, as well as reminding the interviewers to follow the necessary fieldwork protocols.

The prevalence flag indicated whether the cases had disorders or not. The prevalence flag accounted for both positive and negative values calculated for a period of 2 weeks and a cutoff of >+1.5 *z* score for positive prevalence and <−1.5 *Z* score for negative prevalence. The average value was 0.43 (SD 0.11). The highest negative prevalence flag was −2.64 (SD 0.16), whereas the highest positive prevalence flag was 2.47 (SD 0.17). In total, only 21 flags were triggered for the negative prevalence indicator, where the cases included in the calculations had no disorders, and 26 flags were triggered for the positive prevalence flag, where cases had many disorders. When investigated and correlated with other flags, some interviewers with a high negative prevalence also had short interviews and short questions. These interviewers were further investigated and monitored to determine if they were developing certain patterns when interviewing, and corrective actions were taken. Interviewers with high positive prevalence flags exhibited greater skills in convincing the respondents to disclose their mental health disorders.

Most of the short and long interviews were just a few minutes shorter than the cutoff values. The interviewers who cumulated a high number of *short interview length* and *long interview length* flags were monitored. It was found that, in the case of short interviews, the interviewers were reading at a fast pace, but clearly, while those with long interviews were either having technical issues and interruptions, respondents who provided detailed answers or comments, interviewers reading the questionnaire slowly, or breaks requested by the respondent. In the case of a break for >7 minutes, the case was flagged with *long question field time*, which was investigated by tracing the duration of the reported working time, Cisco calling time, ADT time, and Blaise time. In addition, we verified the question where the interviewer was at during the long pause and whether the interviewer left any remarks for that respective question. Some interviewers left notes saying that the respondent took a short break to pray (as Muslims pray 5 times each day) or attend to domestic tasks. In cases where no explanation was provided for a break, the monitoring team observed the interviewers and took corrective actions.

Some of the cases flagged with *high number of completed interviews* and with *high percentage of short field time* (>30% of short questions within 1 interview) were found to have the *short question field time* and *short interview length* flags as well. However, in many instances, the interviewers flagged with a high number of completed cases per day were completing cases at the second or third attempt. This finding meant that previous interviewing sessions were conducted before for the same cases that completed the modules of the survey.

In the case of *short average interview length* flag, the average interview length was calculated for each interview every 2 weeks. If the *z* score for this type of flag was ≤−2, then the interviewer was flagged. We only had 14 *short average interview length* flags. This finding was largely because of the long nature of the WMH Survey, in which the average interview length was 94 minutes for the long version and 64 minutes for the short version.

On the basis of the investigation of triggered flags per interviewer, monitoring, corrective and preventive actions were taken. Although, in most cases, interviewers required refresher training sessions and feedback to improve their performance, several interviewers discontinued work owing to low productivity and a high number of triggered flags.

## Discussion

### Limitations and Challenges

Developing new flags involved determining appropriate cutoffs adapted for the telephone interviewing mode. This was challenging because the previous phone interviews conducted at SESRI were not based on long and complex questionnaires, as was the case for the WMH Qatar Survey. In this regard, some flags required additional calculations to reflect various factors affecting the predetermined values. For instance, after 3 weeks into production, we discovered that many *high number of completes* flags were triggered. After an in-depth investigation and based on the collected data, we found that some interviewers were working extra hours, completing more interviews, and being flagged without taking these factors into consideration. After accounting for extra hours, the number of these flags decreased from 85 to 18 (at the moment of discovery and implementation of the new calculation).

Microsoft Planner posed some difficulties at the start of the survey production phase in relation to documenting the flag investigation process, monitoring observations, and assigning corrective interventions. We initially allocated each flag a task on the Planner. This led to hundreds of tasks generated on a daily basis in the Planner, making it logistically impossible to review and prioritize the flags triggered. We decided to organize the tasks per interviewer instead and to generate weekly frequency for the flags triggered most often (eg, short question field time, multiple field visits, and multiple stem-field visits) rather than daily frequency.

To mitigate the challenges faced during the design, implementation, investigation, and verification phases, the QC team proposed innovative solutions and experimental stages to address various factors affecting the survey QC process. Being one of the first studies in the WMH Survey consortium conducted using CATI mode during the COVID-19 pandemic, no prior evidence was available on how to incorporate QC procedures within these complex surveys. Available, but limited, literature from other parts of the world in relation to using CATI surveys [23] and web surveys [24-28] did not focus on QC procedures. This study aimed to provide an overall framework for implementing similar QC procedures in future large-scale population surveys, including WMH Surveys using CATI technology. Nevertheless, further research and experimentation are necessary for developing sound QC process adapted for large-scale telephone survey interviews.

### Conclusions

QC procedures were essential in conducting the WMH Qatar phone survey in the context of the COVID-19 pandemic. The IT team, the CATI laboratory team, and the research team collaborated to develop strategies to conduct and manage the QC procedures. Switching from the CAPI mode to CATI mode required a complete transformation of the previously established QC system. Initially, the CATI laboratory was based on a centralized call center, and because of the COVID-19 outbreak, the operations had to be switched to a decentralized call center. Various programs were introduced, such as CATI Time Tracker, developed by the SESRI IT team, to accommodate the requirements of phone surveying. Microsoft Planner was also used to provide justifications and document the actions taken by the research team and the CATI laboratory team.

Developing new indicators customized for the phone mode of surveying enhanced the QC process. The flag investigation and verification of the interviewers’ performance shed light on how the interviewers conducted the telephone interviews and the QC standards followed during the data collection phase of the WMH Qatar Survey.

Feasible methods of QC are a high priority for institutions and individuals conducting studies that involve data collection of sensitive and costly population-based surveys. QC measures may present a challenge for remote monitoring of interviewers’ performance. Our efforts to design and implement a survey QC system attempted to solve some of the challenges related to the phone mode of interviewing, especially when conducted remotely for large-scale and costly surveys. We also attempted to build a prototype of the QC system for phone interviewing that can be further used in our local research community. Our findings will help shape future QC procedures in similar studies in which a probability sample is designed under conditions of necessity. This is relevant for survey practice today more than ever because of the increasing demand to collect more quality data at a lower cost and its implications for health policies.

## Abbreviations

ADT: audit trail
CAPI: computer-assisted personal interviewing
CATI: computer-assisted telephone interviewing
CDR: call detail records
CIDI: Composite International Diagnostic Interview
Power BI: Power Business Intelligence
QC: quality control
QCIS: quality control indicator system
SESRI: Social and Economic Survey Research Institute
WMH: World Mental Health

## Data Availability

Data sharing is not applicable to this paper because no data sets were generated or analyzed in this case study.
